# Supplementary material for: Identification of biomarkers for hepatocellular carcinoma based on single cell sequencing and machine learning algorithms
Source: Front Genet. 2022 Oct 24;13:873218. doi: 10.3389/fgene.2022.873218 (PMC9638064; doi:10.3389/fgene.2022.873218)
Supplement: Supplementary file 1 [file Table1.DOCX]

| Rank | Gene | Rank | Gene | Rank | Gene | Rank | Gene | Rank | Gene |
| --- | --- | --- | --- | --- | --- | --- | --- | --- | --- |
| 1 | SPP1 | 11 | AC092580.4 | 21 | APOA2 | 31 | AC133644.2 | 41 | PTGER4 |
| 2 | FCN3 | 12 | AIM1 | 22 | GZMK | 32 | PYHIN1 | 42 | C16orf54 |
| 3 | FCRL6 | 13 | GZMM | 23 | KLRD1 | 33 | KLRG1 | 43 | VPS37B |
| 4 | S1PR5 | 14 | IGHA1 | 24 | RNF125 | 34 | ZAP70 | 44 | GPR65 |
| 5 | CD8A | 15 | SLAMF6 | 25 | INPP5D | 35 | FAM46C | 45 | TBC1D10C |
| 6 | SAA1 | 16 | TRGC1 | 26 | RUNX3 | 36 | PRAP1 | 46 | CACYBP |
| 7 | CD160 | 17 | STAT4 | 27 | CCND3 | 37 | KLRF1 | 47 | GZMH |
| 8 | GPR18 | 18 | SCML4 | 28 | CD69 | 38 | IKZF1 | 48 | ANXA2R |
| 9 | AKNA | 19 | HBB | 29 | PPP1R16B | 39 | TRGC2 | 49 | APBA2 |
| 10 | FCMR | 20 | PLAC8 | 30 | ADGRE5 | 40 | ITK | 50 | GIMAP7 |

**Supplementary Table 1** The top 50 mRMR genes.
